# Diversity of *Phytophthora* Species from Declining Mediterranean Maquis Vegetation, including Two New Species, *Phytophthora crassamura* and *P*. *ornamentata* sp. nov.

**DOI:** 10.1371/journal.pone.0143234

**Published:** 2015-12-09

**Authors:** Bruno Scanu, Benedetto T. Linaldeddu, Antonio Deidda, Thomas Jung

**Affiliations:** 1 Dipartimento di Agraria, Sezione di Patologia vegetale ed Entomologia (SPaVE), Università degli Studi di Sassari, Viale Italia 39, 07100 Sassari, Italy; 2 Phytophthora Research and Consultancy, Am Rain 9, D-83131, Nußdorf, Germany; 3 Center for Mediterranean Bioresources and Food (MeditBio), Laboratory of Molecular Biotechnology and Phytopathology, University of Algarve, Campus de Gambelas, 8005–139 Faro, Portugal; Estacion Experimental de Zonas Aridas - CSIC, SPAIN

## Abstract

The Mediterranean basin is recognized as a global biodiversity hotspot accounting for more than 25,000 plant species that represent almost 10% of the world’s vascular flora. In particular, the maquis vegetation on Mediterranean islands and archipelagos constitutes an important resource of the Mediterranean plant diversity due to its high rate of endemism. Since 2009, a severe and widespread dieback and mortality of *Quercus ilex* trees and several other plant species of the Mediterranean maquis has been observed in the National Park of La Maddalena archipelago (northeast Sardinia, Italy). Infected plants showed severe decline symptoms and a significant reduction of natural regeneration. First studies revealed the involvement of the highly invasive wide-host range pathogen *Phytophthora cinnamomi* and several fungal pathogens. Subsequent detailed research led to a better understanding of these epidemics showing that multiple *Phytophthora* spp. were involved, some of them unknown to science. In total, nine *Phytophthora* species were isolated from rhizosphere soil samples collected from around symptomatic trees and shrubs including *Asparagus albus*, *Cistus* sp., *Juniperus phoenicea*, *J*. *oxycedrus*, *Pistacia lentiscus* and *Rhamnus alaternus*. Based on morphological characters, growth-temperature relations and sequence analysis of the ITS and *cox*1 gene regions, the isolates were identified as *Phytophthora asparagi*, *P*. *bilorbang*, *P*. *cinnamomi*, *P*. *cryptogea*, *P*. *gonapodyides*, *P*. *melonis*, *P*. *syringae* and two new Clade 6 taxa which are here described as *P*. *crassamura* sp. nov. and *P*. *ornamentata* sp. nov. Pathogenicity tests supported their possible involvement in the severe decline that is currently threatening the Mediterranean maquis vegetation in the La Maddalena archipelago.

## Introduction

The Mediterranean basin has been recognised as one of the world’s 25 biodiversity hotspots for priority conservation, accounting for more than 25,000 plant species, around half of which are endemic [1]. In particular, the Tyrrhenian islands and archipelagos are characterized by an extremely high degree of endemism [2]. This characteristic is mainly the outcome of the geological and climatic history during the Cenozoic era, when these territories became a crossroad of taxa from different continents and served as floristic refuges during interglacial periods [3]. The National Park of La Maddalena archipelago (Italy), located between northeast Sardinia and southern Corsica, is considered a micro-hotspot, hosting more than one thousand vascular-plant taxa, 54 being Sardinian endemics [4]. This archipelago comprises 7 major islands and 55 small islets, covering a land area of around 5,134 hectares [5]. While the islets are characterized by rocky and dry sites unable to support woodland or forest, the main islands are covered with dense evergreen forests of *Quercus ilex* and *Juniperus phoenicea*, mixed with several shrub species typical of the Mediterranean maquis such as *Arbutus unedo*, *Cistus* spp. and *Erica arborea* as understorey layer. Different vegetation types, including heath, scrub-heath with or without low trees and thicket, generally consisting of shrub species approximately 1 m or less in height occur in lower-laying sites and sites more exposed to winds, with *A*. *unedo*, *J*. *phoenicea*, *Olea europaea* var. *sylvestris*, *Phillyrea angustifolia* and *Pistacia lentiscus* as the main components [5]. Because of its unique habitats, the archipelago of La Maddalena has been declared as a Site of Community Importance and Special Protection Area for biodiversity conservation (Council Directive 92/43/EEC).

Since 2008, a serious and widespread decline and mortality of *Q*. *ilex* trees has been reported on Caprera Island, the second-largest island of the archipelago [6]. During the intense study of this epidemic event, several *Botryospaeriaceae* and *Phytophthora* spp. were isolated from declining *Q*. *ilex* trees, and *Diplodia corticola* and *Phytophthora cinnamomi* were shown to be the main drivers of this disease [7]. Subsequently, the recently described *Phytophthora parvispora* was recovered from dying and dead plants of *A*. *unedo* on Caprera Island [8]. During these surveys, extensive dieback and mortality of several other plant species typical of the Mediterranean maquis, including *Asparagus albus*, *Cistus* sp., *J*. *phoenicea*, *J*. *oxycedrus*, *P*. *lentiscus* and *Rhamnus alaternus* were observed and further investigations were carried out to establish whether *Phytophthora* species were also associated with these symptomatic trees and shrubs. In a preliminary study, an unexpected array of *Phytophthora* species was recovered, some of them common in forests worldwide and others rarely reported in forests or previously rarely recorded at all [9]. In addition, two groups of isolates could not be assigned to any known species or informally designated taxon of *Phytophthora*.

The main objectives of the present work were: i) to study the diversity of *Phytophthora* species from declining Mediterranean maquis vegetation in the National Park of La Maddalena archipelago; ii) to characterize the isolates of the two putative new *Phytophthora* species in terms of morphology, growth-temperature relationship and phylogenetic position; iii) to assess the aggressiveness to main woody Mediterranean plant species of all *Phytophthora* species obtained in this study. The results of these investigations are presented and the two new taxa described as *P*. *crassamura* sp. nov. and *P*. *ornamentata* sp. nov.

## Material and Methods

### Ethics statement

This study is part of a collaborative project with the National Park of La Maddalena archipelago from where *Phytophthora* species were isolated. Root and soil samples were collected from symptomatic trees and shrubs on the islands of Caprera, Santo Stefano and Spargi for which no specific permissions were required. Our field sampling did not involve endangered or protected species.

### Sampling and isolation

Between May 2012 and November 2013, soil samples (approximately 1 L) including fine roots were collected from around symptomatic trees and shrubs growing in a natural area of about 50 ha on Caprera Island (41°12′N, 9°27′E). Additional samples were collected in April 2014 on Santo Stefano Island (41°11′N, 9°24′E) and Spargi Island (41°14′N, 9°21′E). Main plant species sampled included *A*. *albus*, *J*. *phoenicea*, *P*. *lentiscus* and *R*. *alaternus*. All collected samples were placed in plastic bags, labeled and transported in cool boxes to the laboratory and processed within 24 hours.

To isolate *Phytophthora*, soil and root samples were baited as described by Jung et al. [10]. Soil and roots were flooded with distilled water in a plastic tray to 3cm depth, and juvenile leaves of *Quercus suber* were floated over the water, acting as baits for *Phytophthora*. After 3–5 days, leaves showing dark spots were examined under the microscope (200x magnification) for presence of sporangia. Positive leaves were cut in small pieces and plated onto Synthetic Mucor Agar (SMA) medium [11] supplemented with 50 mL carrot juice and after autoclaving at 121°C for 15 min amended with 0.4 mL of a 2.5% (w:v) aqueous suspension of pimaricin, 3 mL of a 1% (w:v) aqueous solution of rifamycin SV sodium salt, 0.05 g of hymexazol and 0.2 g of ampicillin (all from Sigma-Aldrich). The plates were checked daily under the stereomicroscope and any developing colonies were subcultured on carrot-agar (CA; 16 g agar technical no.3, Oxoid Ltd, Basingstoke, UK, 200 g carrots and 1000 mL distilled water) [12].

Additionally, in spring and autumn of 2013 ponds and streams were baited on Caprera Island following the method of Hüberli et al. [13]. Unwounded young leaves of susceptible species such as *A*. *unedo*, *Hedera helix*, *Pittosporum undulatum*, *Q*. *ilex* and *Q*. *suber* were placed in a mesh raft rigged to float just below the water surface. After 5–8 days, baits were collected and returned to the laboratory to be examined for the presence of necrosis. Isolations of *Phytophthora* were made on SMA as described above.

### *Phytophthora* isolates and culture maintenance

The isolates used in this study are listed in Table 1. Cultures were maintained at 10°C under water in long-term storage at the Culture Collection of the University of Sassari. The ex-type culture of *P*. *megasperma* (CBS 402.72) sourced from the CBS-KNAW Fungal Biodiversity Centre was included for morphological, physiological and phylogenetic comparison.

**Table 1:** Identity, host, location, isolation date and GenBank accession numbers for *Phytophthora* isolates used for morphological, physiological and phylogenetic analyses in this study. n.a., not available.

Collection no.^a^	*Phytophthora* species	Host species	Sample	Location (ecosystem, region, country)	Isolation date	GenBank accession no.	
						ITS	*Cox*1
PH094	*P*. *crassamura*	*Picea abies*	Collar lesion	Nursery, Sardinia, Italy	November, 2011	KP863492	KP863482
CBS 140357, PH138^b^	*P*. *crassamura*	*Juniperus phoenicea*	Rhizosphere soil	Wetland, Sardinia, Italy	May, 2012	KP863493	KP863485
PH170	*P*. *crassamura*	*J*. *phoenicea*	Rhizosphere soil	Wetland, Sardinia, Italy	May, 2012	KP863494	KP863483
PH171	*P*. *crassamura*	*J*. *phoenicea*	Rhizosphere soil	Forest, Sardinia, Italy	March, 2013	KP863495	KP863484
PH172	*P*. *crassamura*	*J*. *phoenicea*	Ponding water	Forest, Sardinia, Italy	March, 2013	n.a.	n.a.
CBS 402.72^b^	*P*. *megasperma*	*Althaea rosea*	Root rot	United States	1931	HQ643275	KP863479
PH178	*P*. *megasperma*	*Castanea sativa*	Rhizosphere soil	Planting, Sardinia, Italy	November, 2013	KP863491	KP863480
PH192	*P*. *megasperma*	*C*. *sativa*	Rhizosphere soil	Planting, Sardinia, Italy	November, 2013	KP863490	KP863481
PH225	*P*. *megasperma*	*C*. *sativa*	Collar lesion	Planting, Sardinia, Italy	November, 2013	n.a.	n.a.
CBS 140647, PH152^b^	*P*. *ornamentata*	*Pistacia lentiscus*	Rhizosphere soil	Wetland, Sardinia, Italy	November, 2012	KP863496	KP863486
PH153	*P*. *ornamentata*	*P*. *lentiscus*	Rhizosphere soil	Wetland, Sardinia, Italy	November, 2012	KP863497	KP863487
PH167	*P*. *ornamentata*	*P*. *lentiscus*	Rhizosphere soil	Forest, Sardinia, Italy	April, 2012	KP863498	KP863488
PH169	*P*. *ornamentata*	*P*. *lentiscus*	Ponding water	Forest, Sardinia, Italy	April, 2012	KP863499	KP863489
P904^c^	*P*. *cinnamomi*	n.a.	n.a.	Australia	n.a.	KC478662	KC609421
CBS 144.22^c^	*P*. *cinnamomi*	*Cinnamomum* sp.	Stripe canker	Plantation, Sumatra	1922	KC478663	KC609419
CBS 132771^c^	*P*. *parvispora*	*Arbutus unedo*	Rotted roots	Nursery, Sardinia, Italy	2008	GU460376	KC609412
CBS 132772^c^	*P*. *parvispora*	*Arbutus unedo*	Collar rot	Planting, Sardinia, Italy	2011	KC478667	KC609413

^a^ Abbreviations of isolates and culture collections: CBS = CBS-KNAW Fungal Biodiversity Centre, Utrecht, Netherlands; PH = culture collection of the University of Sassari; P = Forest Research Phytophthora culture collection, Farnham, UK.

^b^ ex-type culture.

^c^ isolates used for the mating tests.

### Growth rates and morphological characterization

Colony morphologies were characterized from 5-day-old cultures incubated at 20°C in the dark on CA, V8-juice agar (V8A; 100 mL filtered V8 juice, 0.1 g CaCO_3_ and 900 mL distilled water) [14], potato dextrose agar (PDA) and malt extract agar (MEA). Temperature-growth rate studies were undertaken according to Scanu et al. [8]. Each isolate was incubated with three replicates at 5, 10, 15, 20, 25, 30, 35 and 40°C (all ± 0.5°C). Cardinal temperatures were determined by growing the isolates at one-degree intervals in the temperature ranges 25–30, 5–10 and 35–40°C respectively [8].

Measurements and photographs of morphological structures were made at 200x and 400x magnification and recorded using a digital camera Leica DFC495 connected to a Leitz Diaplan compound microscope (Leitz, Germany) and Leica Application Suite imaging software v.4.5.0 (Leica Microsystems, Switzerland). All measured structures were in a mature stage and selected at random. For sporangia measurements four mycelial plugs (10 mm diam.) were cut from the edges of actively growing colonies on V8A, placed in sterile 60 mm Petri dishes and flooded with unsterile pond water and nonsterile soil extract water. Water cultures were incubated at 20–25°C in natural daylight until sporangia were observed. Chlamydospores and hyphal swellings were assessed directly on CA plates if present. Sporangial length (l), breadth (b) and l/b ratio and characteristic features of 50 sporangia, as well as shape and diameters of 50 chlamydospores and hyphal swellings were recorded for each isolate. Gametangia were examined after 3–4 weeks on CA at 20°C. For those isolates that did not produce or only inconsistently produced oogonia in single culture, sexual compatibility type was determined in paired cultures with A1 and A2 mating type tester strains of *P*. *cinnamomi* and *P*. *parvispora* (Table 1) [8]. Fifty gametangia were chosen at random and dimensions and characteristic features of antheridia, oogonia and oospores were measured and recorded at 200x and 400x magnification. Oospore aplerotic index and oospore wall index were calculated according to Dick [15].

### DNA extraction, amplification and sequencing

DNA was extracted from mycelium using the InstaGene Matrix (BioRad Laboratories, Hercules, CA). The Internal Transcribed Spacers of the ribosomal RNA (ITS) and the cytochrome oxidase I (*cox*1) were amplified and sequenced using primers ITS-6 and ITS-4 [16], and FM 84 and FM 83 [17], respectively. PCR conditions and reaction mixture were as described previously [18], with the exception of the amplification conditions for the *cox*1 that consisted of 1 cycle of 95°C for 2 min followed by 35 cycles of 94°C for 40 s, 55°C for 50 s, 72°C for 1 min and a final extension step of 7 min at 72°C. The PCR products were purified using the EUROGOLD gel extraction kit (EuroClone S.p.A.) following manufacturer’s instructions. ITS and *cox*1 gene regions were sequenced in both directions by the BMR Genomics DNA sequencing service (www.bmrgenomics.it). DNA sequence chromatograms were viewed and edited using BioEdit v. 5.0.6 software [19]. All sequences were deposited at GenBank (http://www.ncbi.nlm.nih.gov/) and accession numbers are given in Table 1.

### Phylogenetic analyses

The ITS and *cox*1 sequences of *Phytophthora* species from ITS Clade 6 [20,21] were downloaded from GenBank and combined with the sequences obtained in this study (Table 1). Sequences were aligned with ClustalX v. 1.83 [22], using the default parameters. Phylogenetic analyses of sequence data were implemented using PAUP v.4.0b10 [23] for Maximum-parsimony (MP) analysis and MrBayes v.3.0b4 [24] for Bayesian Inference (BI) analysis as described previously [25]. All phylograms were rooted to *P*. *cinnamomi* (ex-type isolate CBS 144.22). Alignment files and trees are available from TreeBASE 17435 (http://purl.org/phylo/treebase/phylows/study/TB2:S17435?x-access-code=82888203cc926547c3a78c52b3e46c90&format=html).

### Pathogenicity test

Pathogenicity tests were performed following the soil infestation method described by Scanu et al. [8]. In early April 2014, a total of 96 *J*. *phoenicea* and 88 *P*. *lentiscus* seedlings were inoculated with two isolates each of *P*. *crassamura* (PH094 and PH138), *P*. *megasperma* (CBS 402.72 and PH192) and *P*. *ornamentata* (PH152 and PH153), and one isolate each of *P*. *asparagi* (PH118), *P*. *cinnamomi* (PH190), *P*. *bilorbang* (PH121), *P*. *melonis* (PH120) and *P*. *syringae* (PH135). The latter species was not tested on *P*. *lentiscus*. Seedlings were inoculated by adding 20 mL of inoculum per isolate, whereas control plants received 20 mL of the uninoculated mixture. There were eight replicates per each isolate and controls. After four months, plants were visually assessed for symptoms and mortality rate was recorded; then each plant was removed from the pot and the root system gently washed under tap water. Single roots were cut off at the collar, and after scanning, total root length of all the plant root system was measured using the APS Assess 2.0 software (The American Phytopathological Society, USA). The remaining soil was baited following the method described above to determine whether the pathogen was still viable. Re-isolations were also made from necrotic roots and collar tissues using SMA selective medium.

### Statistical analyses

Morphometric and pathogenicity data were analysed by one-way analysis of variance (ANOVA) using Tukey’s HSD test (Honestly Significant Difference) as a post-hoc test (XLSTAT 2008 software). Differences at *P* < 0.05 were considered significant. Analysis of the differences in growth rates between the two new species and *P*. *megasperma* was performed using the Student’s t-test (*P* < 0.01).

### Nomenclature

The electronic version of this article in Portable Document Format (PDF) in a work with an ISSN or ISBN will represent a published work according to the International Code of Nomenclature for algae, fungi and plants, and hence the new names contained in the electronic publication of a PLOS ONE article are effectively published under that Code from the electronic edition alone, so there is no longer any need to provide printed copies.

In addition, new names contained in this work have been submitted to MycoBank from where it will be made available to the Global Names Index. The unique MycoBank number can be resolved and the associated information viewed through any standard web browser by appending the MycoBank number contained in this publication to the prefix http://www.mycobank.org/MB/. The online version of this work is archived and available from the following digital repositories: PubMed Central, LOCKSS.

## Results

### Disease symptoms

**Fig 1:**
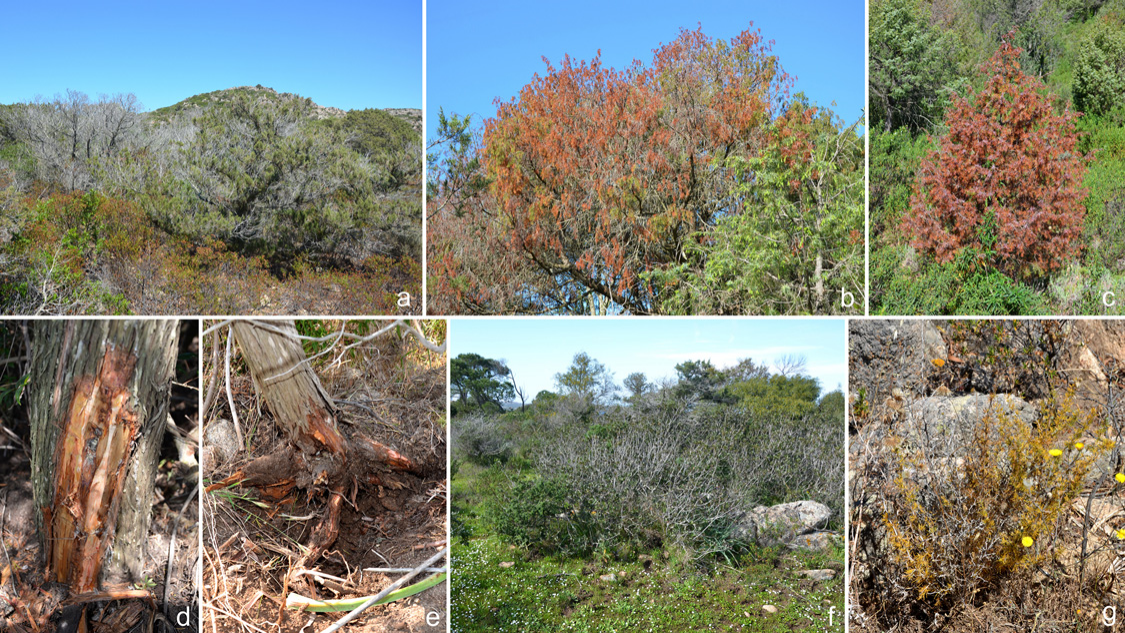
Symptoms of decline on Mediterranean maquis vegetation caused by *Phytophthora* spp.: a. Dead and dying trees of *Juniperus phoeniceae* with abnormal production of epicormic shoots; b. Mature tree of *Juniperus oxycedrus* showing severe wilting and red discoloration; c. Young tree of *J*. *oxycedrus* with red/bronze colour of foliage over the entire crown; d. Basal phloem lesion on a juniper tree extending up from below ground level; e. Collar and root rot on a young juniper tree; f. Extensive dieback and mortality of *Pistacia lentiscus* across site; g. Dieback and wilting of *Asparagus albus*.

Symptoms of decline, dieback and mortality of plant species typical of the Mediterranean maquis were common along slopes downhill of roads and trekking paths in all the three investigated sites within the National Park of La Maddalena archipelago (Fig 1). *Juniperus phoenicea* was severely affected exhibiting a range of symptoms including partial or complete dieback of the crown and abnormal production of epicormic shoots (Fig 1A), dieback, and reddening or browning of drying foliage on dying and recently dead trees (Fig 1B and 1C). Crown symptoms were often associated with extensive losses of both lateral small woody roots and fine roots and the presence of basal phloem lesions extending up from below ground level (Fig 1D). In low-laying areas with seasonal waterlogging collar and root rot were observed on some juniper trees (Fig 1E). In wetlands, also *P*. *lentiscus* showed severe crown thinning and dieback of single branches (Fig 1F), which were associated to root and collar rot. Ground layer species such as *A*. *albus* (Fig 1G) and *Cistus* spp. were also severely affected. Overall, these symptoms were not associated to infections on the upper parts of the plants suggesting that the plants were dying due to a dysfunction and/or destruction of the root system.

### *Phytophthora* species diversity

Nine *Phytophthora* spp. were recovered from 69.3% of the 94 soil samples tested. In total, 96 isolates were obtained from rhizosphere soil samples collected on the three islands from around symptomatic plants belonging to six plant species and from pond and stream baiting on Caprera Island (S1 Fig; Table 2). Most of the isolates conformed morphologically to previously known *Phytophthora* species. *Phytophthora asparagi* and *P*. *bilorbang* were the most common species, with isolation frequencies of 25.5% and 24.5%, respectively (S1 Fig). In contrast, *P*. *melonis*, *P*. *syringae* and *P*. *ornamentata* sp. nov. were isolated at low frequency (less than 5% of samples). *Phytophthora cinnamomi* was the only species recovered from Santo Stefano Island (Table 2) and it was strongly associated with declining *J*. *oxycedrus* and *R*. *alaternus* trees. *Phytophthora asparagi* was the only species associated with *A*. *albus*, whereas *P*. *melonis* and *P*. *syringae* were isolated only from rhizosphere soil samples of *J*. *phoenicea*. *Phytophthora crassamura* was isolated from both *J*. *phoenicea* and *P*. *lentiscus*. *Phytophthora cryptogea* and *P*. *gonapodyides* were recovered only from pond and stream baiting (S1 Fig). Infestations by multiple *Phytophthora* spp. were found in *J*. *phoenicea* and *P*. *lentiscus*. *Phytophthora* spp., and in particular *P*. *asparagi*, *P*. *bilorbang* and *P*. *cinnamomi* were consistently isolated during all seasons.

**Table 2:** *Phytophthora* species recovered from Mediterranean maquis ecosystems in this study, with host, location, number of isolates and GenBank accession numbers of representative specimens.

Species	Host species	Source	Site (island)	No. of isolates	Representative isolates	ITS GenBank Accession no.
*P*. *asparagi*	*Asparagus albus*, *Juniperus phoenicea*, *Pistacia lentiscus*	Rhizosphere soil, water	Caprera, Spargi	24	PH118	KP863492
*P*. *bilorbang*	*Juniperus phoenicea*, *P*. *lentiscus*	Rhizosphere soil, water	Caprera, Spargi	23	PH121	KR011185
*P*. *crassamura*	*J*. *phoenicea*	Rhizosphere soil	Caprera	9	PH138	KP863493
*P*. *cinnamomi*	*Cistus* sp., *Juniperus oxycedrus*, *Rhamnus alaternus*	Rhizosphere soil, water	Caprera, Santo Stefano	18	PH190	KR011189
*P*. *cryptogea*	*P*. *lentiscus*	Water	Caprera	4	PH159	KR011187
*P*. *gonapodyides*	*P*. *lentiscus*	Water	Caprera	4	PH160	KR011188
*P*. *melonis*	*J*. *phoenicea*	Rhizosphere soil	Caprera	2	PH120	KR011184
*P*. *ornamentata*	*P*. *lentiscus*	Rhizosphere soil	Caprera	4	PH152	KP863496
*P*. *syringae*	*J*. *phoenicea*	Rhizosphere soil	Caprera	2	PH135	KR011186

ITS sequence analysis confirmed the morphological identification of all *Phytophthora* species. BLAST searches in GenBank showed 99–100% similarity with reference sequences of representative isolates including those of ex-type cultures (Table 2). Morphologically only isolates of *P*. *asparagi* did not conform to the formal description of this species [26]. Main differences were the prevalence of paragynous instead of amphigynous antheridia, the formation of chlamydospores and a maximum temperature for growth of 35°C. For the remaining two taxa, preliminary morphological examination and ITS sequence analysis showed they could not be assigned to any formally described species or informally designated taxon of *Phytophthora*, hence detailed phylogenetic and taxonomic analyses were carried out.

### DNA phylogeny of the two putative new species

**Fig 2:**
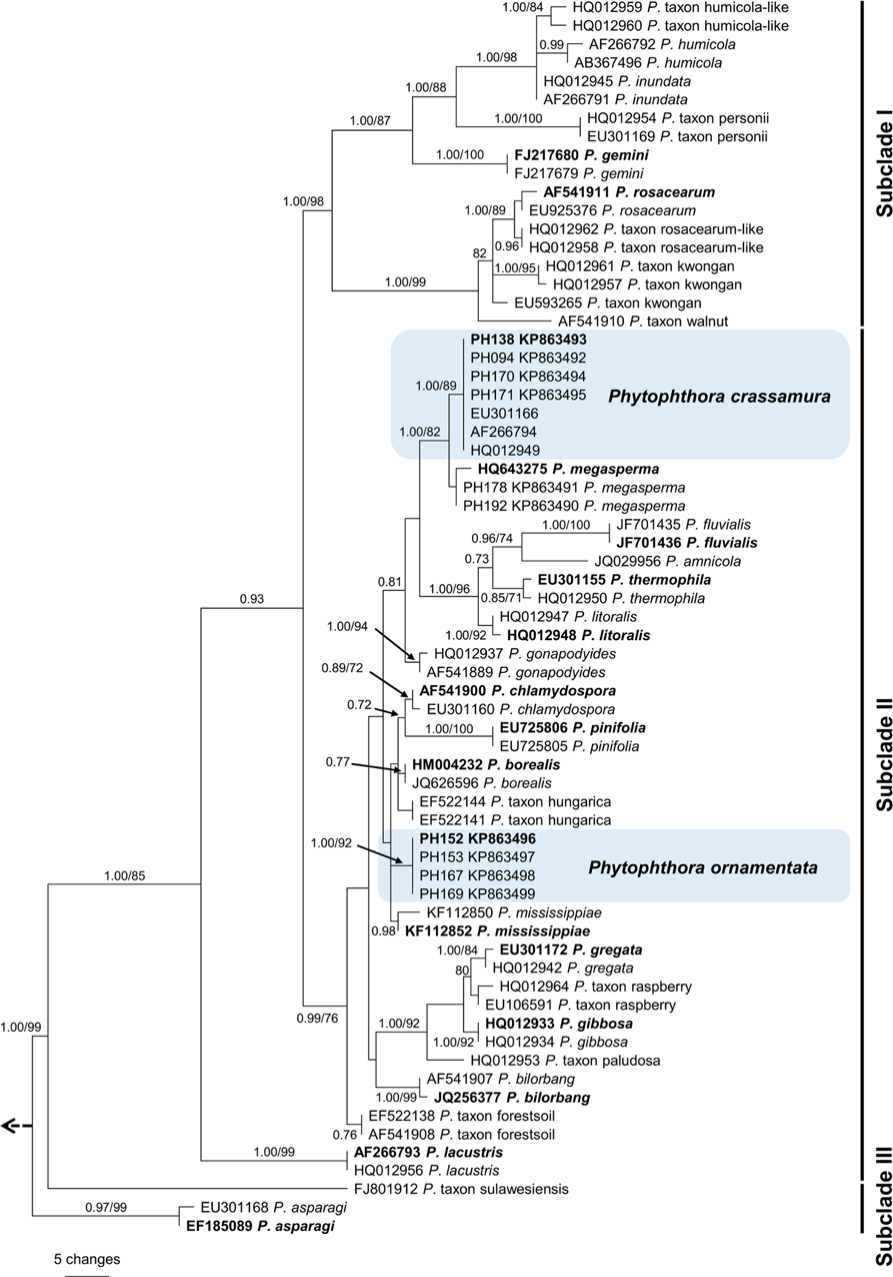
One of the most parsimonious trees based on analysis of rDNA ITS sequence data showing phylogenetic relationships of *Phytophthora* species within ITS Clade 6. Bayesian posterior probabilities (≥ 0.90, left) and bootstrap support values for maximum parsimony (≥ 70%, right) are given at the nodes. Ex-type cultures are in bold. The phylogram is rooted to *Phytophthora cinnamomi* (CBS 14422/KC478663). Sub-clades I–III are indicated on the right.

Phylogenetic analyses of the individual nuclear (ITS) and mitochondrial (*cox*1) datasets resulted in similar overall tree topologies. The aligned datasets for ITS comprised 68 sequences, including seven from *P*. *crassamura* sp. nov. and four from *P*. *ornamentata* sp. nov., with 855 characters of which 149 were parsimony informative. Heuristic searches resulted in 144 most parsimonious trees of 364 steps (CI = 0.68, RI = 0.88). One most parsimonious tree is illustrated in Fig 2 (TreeBASE: 17435). The phylogenetic analysis resolved the three subclades in *Phytophthora* Clade 6 [20,21], accommodating *P*. *crassamura* and *P*. *ornamentata* within subclade II. Isolates of *P*. *crassamura* grouped in a well-supported clade sister to isolates of *P*. *megasperma*. However, *P*. *crassamura* differs from the ex-type culture of *P*. *megasperma* (CBS 402.72) and the two isolates of *P*. *megasperma* from *C*. *sativa* (PH178, PH192) by 3 bp and 5 bp, respectively (Table 3). Three isolates previously designated as *P*. *megasperma* in Australia (DDS 3432, VHS 17183, IMI 389741) by Brasier et al. [20], Burgess et al. [27] and Jung et al. [21] clustered together with isolates of *P*. *crassamura*. Isolates of *P*. *ornamentata* formed a distinct group in the ITS tree within a closely related cluster of six taxa that also includes *P*. *chlamydospora*, *P*. *pinifolia*, *P*. *borealis*, *P*. *mississippiae* and *P*. taxon hungarica. The most closely related species to *P*. *ornamentata* is *P*. *mississippiae*, which differs by 5–8 bp while the other four taxa differ by 9 bp.

**Table 3:** Morphological characters, morphometric data and temperature-growth relations on Carrot Agar of closely related *Phytophthora* species from Clade 6. n.a., not available.

	*P*. *crassamura*	*P*. *megasperma*	*P*. *ornamentata*	*P*. *mississippiae*	*P*. *gibbosa* ^1^
Number of isolates examined	5	4	4	Yang et al. (2013)	Jung et al. (2011)
Sporangia	Ovoid, obpyriform, nonpapillate	**Elongated**, obpyriform, limoniform, nonpapillate	Ovoid, obpyriform, ellipsoid, nonpapillate	Ovoid, obpyriform, nonpapillate, **some semipapillate**	Ovoid, ellipsoid, nonpapillate, **some semipapillate**
Length x breath mean (μm)	60.3 ± 6.0 × 37.4 ± 3.6	74.7 ± 9.5 × 32.0 ± 2.0	59.5 ± 6.2 × 36.8 ± 4.6	60.4 ± 6.0 × 31.3 ± 4.5	48.8 ± 9.6 × 30.8 ± 5.4
Range of isolate means (μm)	54.8–65.4 × 32.4–41.7	68.2–81.4 × 30.1–35.0	42.8–74.5 × 28.5–46.0	47.3–77.3 × 20.4–43.3	44.8–52.2 × 27.9–33.0
Total range (μm)	48.2–72.8 × 22.6–52.4	59.7–89.1 × 26.3–36.6	38.6–78.8 × 21.2–53.4	n.a.	24.8–71.1 × 17.4–48.0
Length/breath ratio	1.6 ± 0.1	**2.3 ± 0.2**	1.6 ± 0.1	1.96	1.58 ± 0.15
Direct germination after 24-48h^2^	**+++**	++	+	n.a.	n.a.
Proliferation	Internal nested and extended, external	Internal nested and extended, external	Internal nested and extended, **never external**	Internal nested and extended, external	Internal extended, external, **never nested**
Hyphal swellings	Globose, elongated, catenulate	Globose, elongated, catenulate	Globose, elongated, catenulate	Globose, elongated, catenulate	Subglobose, elongated, **never catenulate**
Breeding system	Homothallic	Homothallic	**Homothallic**	**Heterothallic**	Homothallic
Oogonia	Smooth-walled	Smooth-walled	**Ornamented**	Ornamented	Ornamented, **smooth**
Mean diameter (μm)	45.4 ± 2.8	41.9 ± 4.4	34.2 ± 4.0	38.2	38.1 ± 5.4
Range of isolate means (μm)	43.8–47.1	39.8–43.5	31.8–38.1	n.a.	36.6–39.7
Diameter range (μm)	35.1–51.6	31.1–49.6	27.6–42.3	n.a.	27.0–49.9
Oospores	**Highly aplerotic**	Slightly plerotic	Slightly aplerotic	Plerotic	Always aplerotic
Mean diameter (μm)	38.2 ± 2.6	36.4 ± 4.0	34.2 ± 4.0	34.0	31.4 ± 4.6
Diameter range (μm)	27.8–44.8	25.2–46.9	26.8–43.4	n.a.	18.9–39.4
Wall thickness (μm)	**4.8 ± 0.6**	3.0 ± 0.9	**4.3 ± 0.8**	n.a.	3.17 ± 0.69
Oospore wall index	**0.57 ± 0.04**	0.41 ± 0.08	**0.63 ± 0.08**	n.a.	0.49 ± 0.06
Abortion rate of isolates (%)	26–44	12–25	13–22	n.a.	16–37
Antheridia	Mostly paragynous	Mostly paragynous	**Mostly paragynous**	Amphigynous	Amphigynous
Length x breath mean (μm)	12.5 ± 2.0 × 11.5 ± 1.5	12.6 ± 1.1 × 11.4 ± 0.7	15.7 ± 2.0 × 13.7 ± 2.6	19.5 × 14.3	13.6 ± 2.4 × 14.0 ± 2.0
Total range (μm)	8.3–15.8 × 7.6–13.9	8.2–15.6 × 7.8–13.6	10.6–19.8 × 9.3 ± 17.5	n.a.	10.6–24.9 × 7.6–17.8
Maximum temperature (°C)	32.5–< 35	30–32.5	32.5–< 35	35	32.5–< 35
Optimum temperature (°C)	25	25	25	25	30
Growth rate at optimum (mm/day)	7.1 ± 0.1	6.2 ± 0.1	6.0 ± 0.2	n.a.	6.3 ± 0.3
Growth rate at 20°C (mm/day)	5.8 ± 0.2	5.2 ± 0.1	5.0 ± 0.1	n.a.	5.2 ± 0.1

^1^Morphological characters and temperature-growth rates of *Phytophthora gibbosa* were examined on V8A.

^2^Presence of sporangia with direct germination: +++, abundant; ++, frequent; +, occasional.

**Fig 3:**
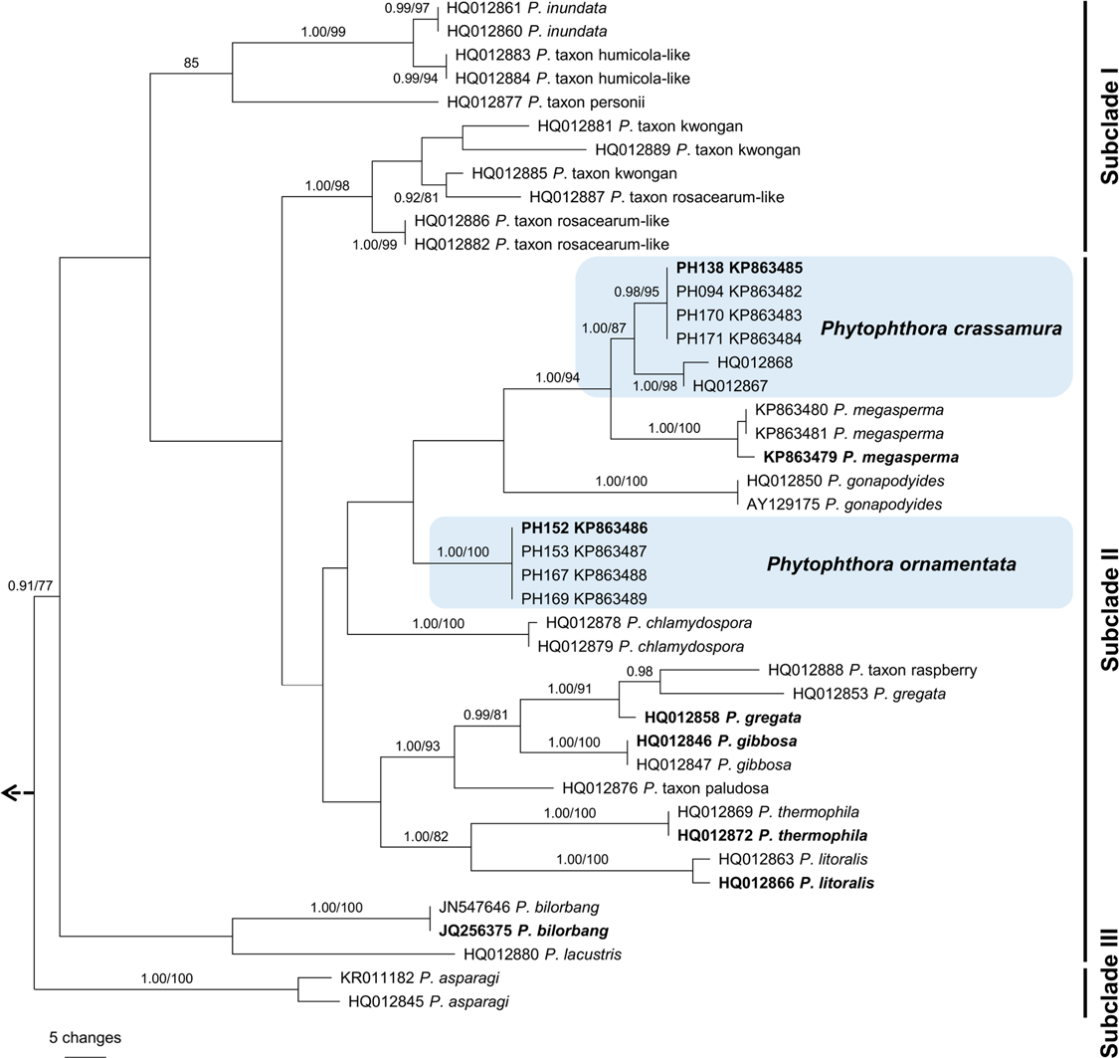
One of the most parsimonious trees based on analysis of mitochondrial DNA *cox*1 sequence data showing phylogenetic relationships of *Phytophthora* species within ITS Clade 6. Bayesian posterior probabilities (≥ 0.90, left) and bootstrap support values for maximum parsimony (≥ 70%, right) are given at the nodes. Ex-type cultures are in bold. The phylogram is rooted to *Phytophthora cinnamomi* (CBS 144.22/KC609419). Sub-clades I–III are indicated on the right.

The mtDNA *cox*1 dataset (44 sequences) consisted of 1127 characters, of which 170 were parsimony informative. Heuristic searches resulted in 22 most parsimonious trees of 593 steps (CI = 0.52, RI = 0.76). One most parsimonious tree is presented in Fig 3 (TreeBASE 17435). As in the ITS analysis both *P*. *crassamura* and *P*. *ornamentata* isolates fell within subclade II of Clade 6. Amongst isolates of *P*. *crassamura* there was considerably higher intraspecific variability in the *cox*1 than in the ITS sequences. The two isolates from Australia identified as *P*. *crassamura* formed a distinct lineage (Fig 3) which differed by 12 to 15 bp from the isolates obtained in this study (S1 Table). Twenty-three fixed polymorphisms distinguished *cox*1 sequences of *P*. *crassamura* from its closest relative *P*. *megasperma* (S1 Table). All isolates of *P*. *ornamentata* had identical *cox*1 sequences and grouped in a strongly supported clade.

### Colony morphology, cardinal temperatures and growth rates

**Fig 4:**
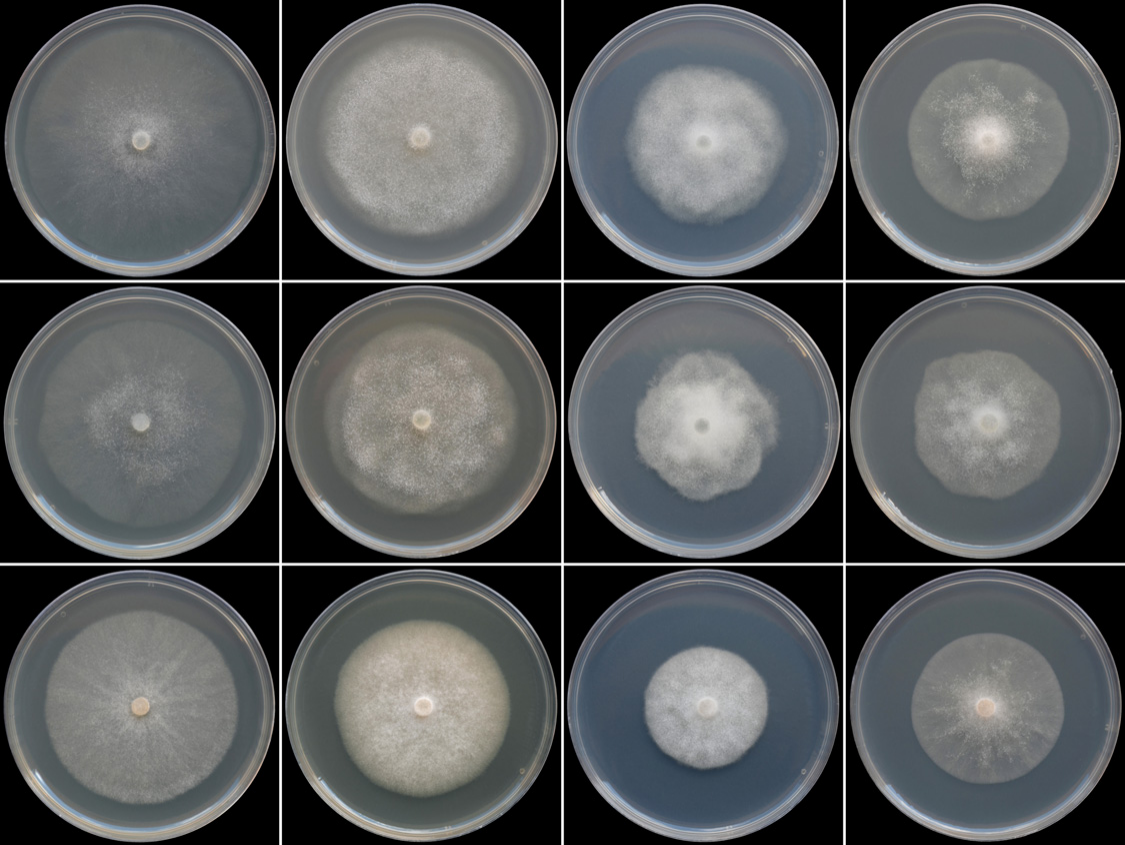
Colony morphology of *Phytophthora crassamura* isolate CBS 140357, *P*. *megasperma* isolate CBS 402.72 and *P*. *ornamentata* isolate CBS 140647 (from top to bottom) after 5 days growth at 20°C on Carrot Agar, V8-Agar, Potato-Dextrose Agar and Malt Extract Agar (from left to right).

Colony growth patterns of each one isolate of *P*. *crassamura* (ex-type culture CBS 140357), *P*. *megasperma* (ex-type culture CBS 402.72) and *P*. *ornamentata* (ex-type culture CBS 140647) are shown in Fig 4. *Phytophthora crassamura* and *P*. *megasperma* formed similar colonies on the four different types of media. On CA both species had faintly striate and mostly submerged colonies, while on the other media colonies were felty (V8A), woolly (PDA) or with limited aerial mycelium around the inoculum plug (MEA) with slightly irregular and not sharply defined edges and faintly petaloid patterns. Conversely, *P*. *ornamentata* formed colonies with sharp margins, slightly radiate to striate with limited aerial mycelium on CA and MEA, and uniform with dense felty mycelium on V8A and PDA. There were no variations in colony morphology. All three species showed slow growth on MEA and PDA.

Cardinal temperatures for growth on CA for *P*. *crassamura*, *P*. *megasperma* and *P*. *ornamentata* isolates are summarized in Table 3, and the temperature–growth rate curves are shown in S2 Fig. All four isolates of *P*. *crassamura* tested showed similar temperature-growth rates and identical cardinal temperatures, with a minimum below 5°C and a maximum between 32.5 and 35°C. None of the isolates grew at 35°C. The average radial growth rate at the optimum temperature of 25°C was 7.1 mm day^-1^. Compared to *P*. *crassamura*, isolates of *P*. *megasperma* had a lower maximum temperature (30–32.5°C) and slower growth rates above 17°C. Radial growth rate of *P*. *megasperma* was 6.2 mm day^-1^ at 25°C. *Phytophthora ornamentata* isolates had similar cardinal temperatures as *P*. *crassamura*. Optimum temperature for growth was 24–25°C with a radial growth rate of 6.0 mm day^-1^.

### Taxonomy

**Fig 5:**
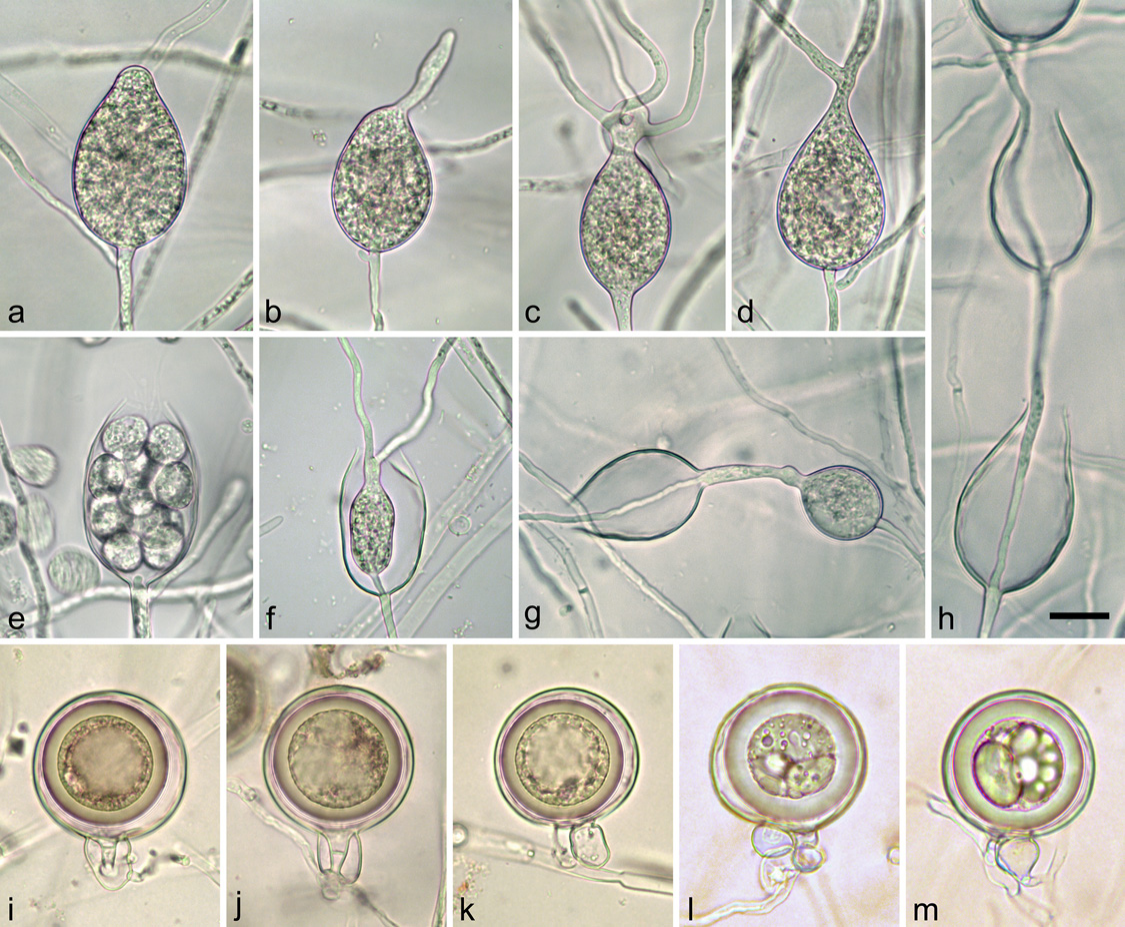
Morphological structures of *Phytophthora crassamura* formed on V8 Agar; a-h. Sporangia produced in nonsterile soil extract water; a. Mature non-papillate, obpyriform, persistent sporangium with an external proliferation just below the base of the sporangium; b-d. Sporangia showing direct germination of sporangiophores; e. Ovoid sporangium releasing individual zoospores; f. Empty sporangium with nested and extended proliferation; g. Sporangium with internal proliferation and intercalary hyphal swelling close to the base; h. Internal extended proliferation; i-m. Mature oogonia with aplerotic and thick-walled oospores; i-j. Oogonia with amphigynous antheridia; k-m. Oogonia with paragynous antheridia; l-m. Aborted oospores with extremely thick wall. Scale bar = 20μm.

***Phytophthora crassamura*** B. Scanu, A. Deidda & T. Jung sp. nov. (Fig 5).

MycoBank: MB 814006

*Etymology*: Name refers to the thick-walled oospores (‘crassa’ = ‘thick’ and ‘mura’ = ‘wall’).

*Typus*: Italy, Sardinia. Isolated from rhizosphere soil of a dying *Juniperus phoenicea* tree. Collected: B. Scanu, 2012; CBS H-22392 (holotype, dried culture on CA, Herbarium CBS-KNAW Fungal Biodiversity Centre), CBS 140357 = PH138 (ex-type culture). ITS and *cox*1 sequence GenBank KP863493 and KP863485, respectively.

*Additional specimens*: Italy, Sardinia. Isolated from collar lesion of a declining nursery plant of *Picea abies*. Collected: B. Scanu, 2011; PH094. Italy, Sardinia. Isolated from rhizosphere soil of a declining *J*. *phoenicea* in a natural forest. Collected: B. Scanu, 2012; PH170. Italy, Sardinia. Isolated from rhizosphere soil of a declining *J*. *phoenicea* in a natural forest. Collected: B. Scanu, 2012; PH171.

**Fig 6:**
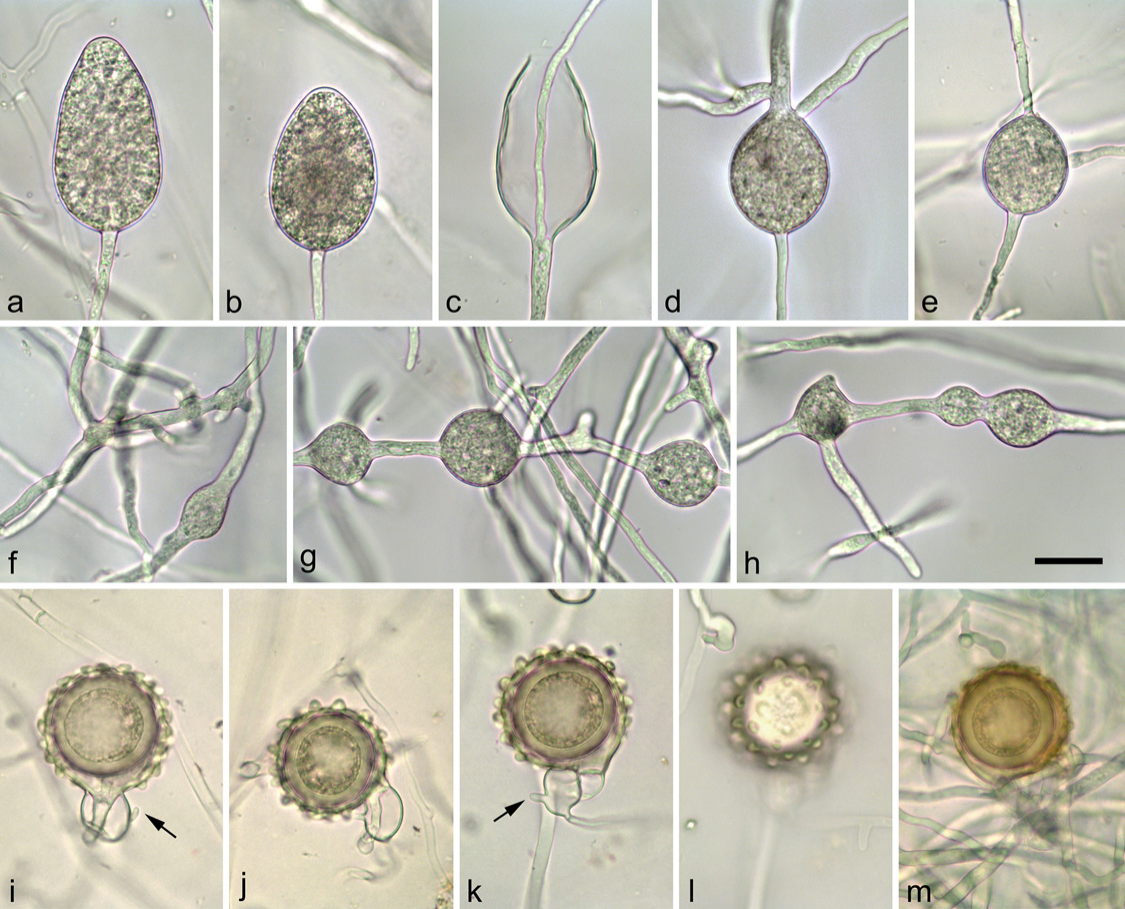
Morphological structures of *Phytophthora ornamentata* formed on V8 Agar; a-e. Sporangia produced in nonsterile soil extract water; a-b. Mature non-papillate, obpyriform to ovoid, persistent sporangia; c. Empty, elongated, ovoid sporangium showing both internal extended proliferation and formation of an additional basal sporangiophore; d-e. Sporangia that failed to form a basal septum and continue to grow with hyphae from the apex of the sporangia, which de facto have the status of hyphal swellings; f. Irregular catenulate hyphal swellings; g-h. Globose to subglobose hyphal swellings with radiating hyphae; i-m. Mature ornamented oogonia and antheridia with finger-like projections (arrow); i-j. Oogonia with amphigynous antheridia; k. Oogonium with paragynous antheridium; k-l. Same oogonium showing the ornamented protuberances on the surface of the oogonial wall; m. Mature bronze-brown oogonia. Scale bar = 20μm.

*Phytophthora crassamura* produces sporangia in both solid media (CA) and more abundantly in liquid culture (soil extract water) after 24 hours of incubation at 20°C. Nonpapillate and noncaducous sporangia (Fig 5A–5H) develop terminally on simple, mostly unbranched sporangiophores. They are, commonly ovoid and obpyriform (Fig 5A and 5B), sometimes with a distorted and pointed apex. Direct germination of sporangia, often with multiple hyphae, through the apex is frequently observed (Fig 5B, 5D and 5F). Some sporangia do not form a basal septum and continue growing at the apex thus being functionally reduced to the status of hyphal swellings (Fig 6C). Zoospores are usually discharged in the water (Fig 5E), or sometimes germinate inside the sporangium. Sporangia proliferate internally in both a nested (Fig 5F) and extended way (Fig 5G and 5H). Chains of proliferating sporangia along the same sporangiophore are common (Fig 5H). External proliferation is also frequent, with new sporangiophores often emerging just below the mature sporangium (Fig 5A, 5B and 5D). Sporangial l x b dimensions of *P*. *crassamura* are 60.3 ± 6.0 × 37.4 ± 3.6 μm (mean ± SD) with an l/b ratio of 1.6 (Table 3). As a comparison, sporangia of the closely related *P*. *megasperma* are mostly elongated, obpyriform and limoniform and on average considerably larger. Sporangial dimensions of four isolates of *P*. *megasperma*, including the ex-type culture (CBS 402.72), averaged 74.7 ± 9.5 × 32.0 ± 2.0 μm, with l/b ratio 2.3 (Table 3). In both species catenulate, globose to subglobose hyphal swellings are formed in nonsterile soil extract water, while chlamydospores were never observed.

All *P*. *crassamura* isolates are homothallic and readily produce gametangia in single culture at 20°C on CA. Oogonia are borne both laterally (Fig 5I and 5K) and terminally (Fig 5L and 5M), and have smooth wall and globose to subglobose shape (Fig 5I–5M). Mean diameter is 45.4 ± 2.8 μm (Table 3). Oospores mature within 14–21 days and are always aplerotic (plerotic index = 59.6%), averaging 38.2 ± 2.6 μm. Oospore wall is extremely thick (4.8 ± 0.6 μm), often turning golden-brown with age. The oospore wall index is 0.57 ± 0.04 (Table 3). The percentage of oogonial or oospore abortion varies between 26–44% amongst isolates. Antheridia are mostly rounded, either paragynous (70%; Fig 5K–5M) or amphigynous (30%; Fig 5I and 5J), with mean dimensions of 12.5 ± 2.0 × 11.5 ± 1.5 μm. Oogonia of *P*. *megasperma* are also smooth-walled, with globose to subglobose shape, and a mean diameter of 41.9 ± 4.4 μm (Table 3). Oospores mature within 21–27 days and are slightly plerotic (plerotic index = 65.4%), averaging 36.4 ± 4.0 μm. Oospore walls are thinner than in *P*. *crassamura* averaging 3.0 ± 0.9 μm with a wall index of 0.42 ± 0.09 (Table 3). The percentage of oogonial or oospore abortion is low (18%). Antheridia are mostly rounded, both paragynous (80%) and amphigynous (20%), with mean dimensions of 12.6 ± 1.1 × 11.4 ± 0.7 μm.

*Notes*. In previous studies, *P*. *crassamura* was referred to as *P*. *megasperma* [20,21,27,28]. Seven accessions of *P*. *megasperma* isolates at NCBI GenBank matched the sequence data of *P*. *crassamura*, however they are not linked to any formal publication. Key differences between *P*. *crassamura* and *P*. *megasperma* are: (i) the higher maximum temperature for growth and faster growth rates at most temperatures in *P*. *crassamura*; (ii) highly aplerotic oospores in *P*. *crassamura vs*. slightly plerotic oospores in *P*. *megasperma*; (iii) extremely thick-walled oospores with a higher oospore wall index in *P*. *crassamura*; (iv) and much more abundant production of oogonia in *P*. *crassamura*. Phylogenetically, *P*. *crassamura* differs from *P*. *megasperma* by a minimum of 3 and 23 fixed polymorphisms in ITS and *cox*1 sequences, respectively (S1 Table).

***Phytophthora ornamentata*** B. Scanu, B. Linaldeddu & T. Jung sp. nov. (Fig 6).

MycoBank: MB 814009

*Etymology*: Name refers to the ornamentation of the oogonial wall.

*Typus*: Italy, Sardinia. Isolated from rhizosphere soil collected beneath declining *Pistacia lentiscus*. Collected: B. Scanu, 2012; CBS H-22393 (holotype, dried culture on CA, Herbarium CBS-KNAW Fungal Biodiversity Centre), CBS 140647 = PH152 (culture ex-type). ITS and *cox*1 sequence GenBank KP863496 and KP863486, respectively.

*Additional specimens*: Italy, Sardinia. Isolated from rhizosphere soil of a dying *P*. *lentiscus* shrub in a natural area. Collected: B. Scanu, 2012; PH153. Italy, Sardinia. Isolated from rhizosphere soil of a declining *P*. *lentiscus* shrub in a natural area. Collected: B. Scanu, 2013; PH167. Italy, Sardinia. Isolated from rhizosphere soil of a declining *P*. *lentiscus* shrub in a natural area. Collected: B. Scanu, 2013; PH169.

*Phytophthora ornamentata* produces sporangia only rarely in solid media (CA) but readily though not abundantly when CA plugs are flooded with nonsterile soil extract water after 24–48 hours of incubation at 20°C. Sporangia are borne terminally or occasionally intercalary. They are nonpapillate and persistent (Fig 6A–6C), commonly obpyriform (Fig 6A) or ovoid (Fig 6B), and less frequently ellipsoid. Sporangia proliferate internally in both a nested and extended way (Fig 6D), whereas external proliferation could never be observed. Chains of proliferating sporangia along the same sporangiophore are frequent. Many sporangia fail to form a basal septum and continue to grow with a hypha from the apex of the sporangium which *de facto* has the status of a hyphal swelling (Fig 6D and 6E). Sporangial l x b dimensions of *P*. *ornamentata* average 59.5 ± 6.2 × 36.8 ± 3.7 μm (mean ± SD) with an l/b ratio of 1.6 ± 0.1 (Table 3). Catenulate, intercalary, globose to subglobose (Fig 6F) and irregular (Fig 6G and 6H) hyphal swellings are abundantly produced by most isolates in liquid culture. Chlamydospores are not formed in any agar media used in this study.

*Phytophthora ornamentata* is homothallic and readily produces gametangia in single culture at 20°C on CA. Oogonia are borne laterally (Fig 6I) or terminally (Fig 6J and 6K). They are globose to subglobose, usually ornamented with warty protuberances on the surface of the oogonial wall (Fig 6I–6L), turning golden-brown to bronze while ageing (Fig 6M). Some oogonia have a tapering base (Fig 6I) while others are distinctly comma-shaped (Fig 6J). Oogonial diameters average 34.2 ± 4.0 μm (Table 3). Oospores mature within 4–5 weeks; they are slightly aplerotic (plerotic index = 64.2%), averaging 29.4 ± 3.3 μm. Oospores are thick-walled (4.3 ± 0.8 μm), with an oospore wall index averaging 0.63 ± 0.08 (Table 3). Oogonial or oospore abortion is low (16%). Antheridia are mostly rounded, both paragynous (80%; Fig 6J and 6K) and amphigynous (20%; Fig 6I), with mean dimensions of 15.7 ± 2.0 × 13.7 ± 2.6 μm. Short hyphal projections are often formed at the base of the antheridia (Fig 6I–6K).

*Notes*. Phylogenetically, *P*. *ornamentata* resides in a strongly supported terminal cluster within subclade II of major Clade 6. *Phytophthora ornamentata* can be easily distinguished from other related species by ITS and *cox*1 sequence data, and by a combination of morphological and physiological characters, of which the most significant ones are highlighted in Table 3. *Phytophthora ornamentata* produces ornamented oogonia like the closely related *P*. *gibbosa* and *P*. *mississippiae*, but can be separated from those by its paragynous antheridia, whereas both *P*. *gibbosa* and *P*. *mississippiae* produce exclusively amphigynous antheridia. In addition, *P*. *ornamentata* differs from *P*. *mississippiae* by its homothallic breeding system whereas *P*. *mississippiae* is self-sterile, and from *P*. *gibbosa* by having lower optimum and maximum temperatures for growth. Both *P*. *mississippiae* and *P*. *gibbosa* produce both nonpapillate and semipapillate sporangia, while those of *P*. *ornamentata* are exclusively nonpapillate.

### Pathogenicity test

Based on mortality rates after four months, *P*. *asparagi* and *P bilorbang* were the most aggressive pathogens on *J*. *phoenicea*, killing 50% and 37.5% of seedlings, respectively. Plant deaths occurred also on seedlings inoculated with *P*. *cinnamomi* and *P*. *syringae* (both with average mortality of 25%), while the remaining *Phytophthora* spp. only caused wilting and chlorosis. Seedlings of *P*. *lentiscus* were also susceptible, with almost all *Phytophthora* species being able to kill plants. All seedlings inoculated with *P*. *cinnamomi* died after three months. Plant deaths above 50% were also observed on seedlings inoculated with *P*. *asparagi*, *P*. *crassamura*, *P*. *bilorbang*, *P*. *melonis* and *P*. *ornamentata*. Control plants did not show any aboveground symptoms and exhibited faster growth.

**Fig 7:**
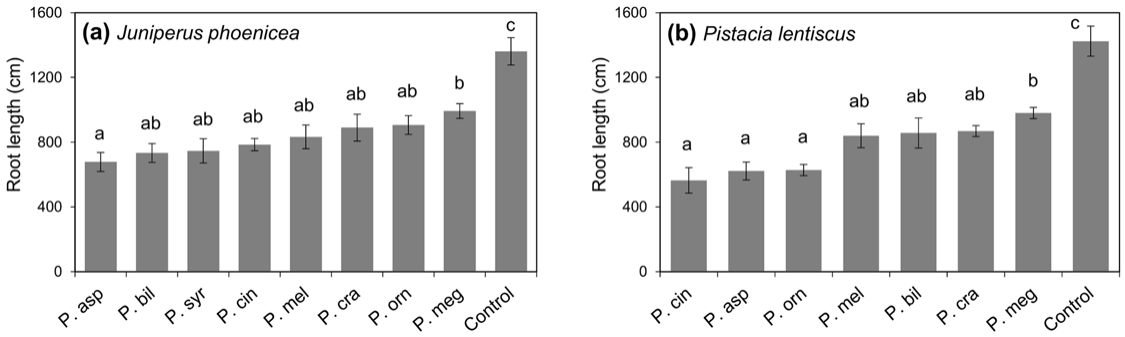
Mean total root length of 1-year-old seedlings of *Juniperus phoenicea* (a) and *Pistacia lentiscus* (b) after 4 months growth in soil infested with *Phytophthora* spp. obtained in this study. Different letters above bars indicate significant differences based on Tukey’s HSD test (*P* = 0.05). Bars represent standard errors.

All *Phytophthora* species tested caused a significant reduction of total root length of both *J*. *phoenicea* and *P*. *lentiscus* (Fig 7A and 7B). Mean total root length was consistently higher in control seedlings than in seedlings infected with *Phytophthora*. On *J*. *phoenicea*, all *Phytophthora* species were able to cause a significant reduction of total root length (*P* < 0.0001). *Phytophthora asparagi* caused significantly higher root reduction than the other *Phytophthora* species for which Tukey’s test revealed no significant differences in total root length of inoculated seedlings (Fig 7A). *Phytophthora cinnamomi*, *P*. *asparagi*, and *P*. *ornamentata* were the most aggressive root pathogens on *P*. *lentiscus* (*P* < 0.0001), causing more than 60% reduction of total root length (Fig 7B). All *Phytophthora* isolates were re-isolated from both necrotic roots and soil. No *Phytophthora* isolates were recovered from control seedlings.

## Discussion

A total of nine *Phytophthora* taxa were isolated from rhizosphere soil samples collected from declining Mediterranean maquis vegetation and river catchments in the National Park of La Maddalena archipelago. These included species common in natural and forest ecosystems in Europe such as *P*. *cinnamomi*, *P*. *cryptogea*, *P*. *gonapodyides* and *P*. *syringae* and the less widespread species *P*. *asparagi*, *P*. *bilorbang* and *P*. *melonis*. In addition, two taxa did not correspond to any known species and are described here as new species, *P*. *crassamura* sp. nov. and *P*. *ornamentata* sp. nov.

The most common species encountered was *P*. *asparagi*, which was isolated from rhizosphere soil beneath declining *A*. *albus*, *J*. *phoenicea* and *P*. *lentiscus* in two separated islands. Apart from *A*. *albus*, Koch’s postulates were fulfilled for the latter two species and these represent new records of *P*. *asparagi* from these host plants worldwide. Although *P*. *asparagi* was described causing water-soaked lesions on roots and shoots of *Asparagus officinalis* in Southwest Michigan, USA [29,30], it had already been isolated from *A*. *officinalis* 20 years earlier in Italy by Cacciola et al. [31]. *Phytophthora asparagi* has also been reported from members of the *Agavaceae* (*Agave*, *Yucca* and *Furcraea*) and from *Aloe* sp. at the Royal Botanic Gardens in Melbourne (Australia) [26] and in Italy causing bud and heart rot of *Agave attenuata* [32]. Interestingly, the Sardinian isolates from *A*. *albus*, *J*. *phoenicea* and *P*. *lentiscus* showedseveral substantial differences to the description provided by Granke et al. [30], in particular a higher maximum temperature for growth (35°C vs <30°C), presence of chlamydospores and a prevalence of paragynous antheridia.

The recently described *P*. *bilorbang* was also isolated with high frequency from both *J*. *phoenicea* and *P*. *lentiscus*. This species, previously informally designated as *P*. taxon oaksoil [20], was formerly isolated from rhizosphere soil and roots of declining forest trees in France [33], streams in Oregon [34] and declining European blackberry (*Rubus anglocandicans*) in Western Australia [35]. Recently, *P*. *bilorbang* has been isolated from *Alnus glutinosa* leaves close to river water in a remote forest in Sardinia [25]. In the present study, *P*. *bilorbang* was one of the most frequent *Phytophthora* species isolated from river catchments and seasonal ponding water. This is consistent with all previous records, suggesting that this species is well adapted to aquatic environments where it acts as a saprotroph of leaf debris and occasionally as an opportunistic pathogen [34, 35]. The biology and in particular the breeding strategy of this *Phytophthora* species has been debated [36]; in the formal description, *P*. *bilorbang* was shown to be fully homothallic [35] whereas isolates with identical ITS sequences obtained from France and Oregon [20,34] were shown to be sterile. In agreement with Aghighi et al. [35] the isolates of *P*. *bilorbang* from the present study were abundantly self-fertile.

Like *P*. *asparagi*, *P*. *melonis* had previously been associated only with agriculture and the finding of *P*. *melonis* in the rhizosphere of two adjacent *J*. *phoenicea* trees on Caprera Island represents the first record of this species from a natural environment but also from Europe and *J*. *phoenicea*. *Phytophthora melonis*, which is conspecific to *P*. *sinesis*, causes a severe disease of members of the *Cucurbitaceae* in Japan, mainland China, Taiwan, Iran, Egypt, Turkey and India [37]. In addition to cucumber, *P*. *melonis* infects other cucurbits such as *Cucurbita pepo*, *Cucumis melo*, *Benincasa hispida* [38], and *Trichosanthes dioica* [39]. It has also been reported on *Pistacia vera* causing blight, dieback, root rot, foot rot and crown rot resulting in gummosis [28].

Three species identified as *P*. *cinnamomi*, *P*. *cryptogea* and *P*. *gonapodyides* had already been reported associated with declining holm oak trees on Caprera Island [7]. *Phytophthora cinnamomi* has a cosmopolitan distribution and is notorious for its involvement in the severe dieback epidemics threatening Eucalypt forests, woodlands and heathlands across Australia, chestnut and oak forests in North America and Europe and many other forest and crop trees worldwide [14,40–42]. Most of these epidemics occur in Mediterranean climate and the adaptations enabling survival of *P*. *cinnamomi* during the hot and dry summers have recently been elucidated [43]. This exotic pathogen is also well established in Sardinian forests, in particular associated with severe mortality of cork oak trees [44]. Significant reductions of the root system in the pathogenicity tests on *J*. *phoenicea* and *P*. *lentiscus* showed that *P*. *cinnamomi* has the potential to threaten the native Mediterranean maquis vegetation. This is supported by recent scattered outbreaks of *P*. *cinnamomi* in other juniper stands in Sardinia. The other two species, *P*. *cryptogea* and *P*. *gonapodyides*, were only recovered from streams and ponding water. Both species are generally encountered in Mediterranean forest ecosystems [45,46]; however, they were never associated with Mediterranean maquis vegetation.

Also *P*. *syringae*, which was isolated from two juniper trees on Caprera Island, has already been reported from forest trees in Italy, apparently without causing disease [46]. Compared to the other *Phytophthora* species sampled in this study, *P*. *syringae* has a lower maximum temperature for growth (around 25°C), a character considered typical of *Phytophthora* species from cool temperate regions. However, Pérez-Sierra et al. [45] suggested that homothallic species with thick-walled oospores like *P*. *syringae* might be able to survive severe summer droughts in a dormant state and become active during the mild and wet winter season typical of the Mediterranean climate.

Two previously unknown *Phytophthora* spp. associated with declining *J*. *phoenicea* and *P*. *lentiscus* trees and shrubs on Caprera Island were identified in this study. *Phytophthora crassamura* and *P*. *ornamentata* are easily distinguished from related or morphologically similar species based on both ITS and *cox*1 sequence data, as well as by a range of morphological and physiological criteria (see Notes and Table 3). Phylogenetic analyses of both the nuclear ITS rDNA and mitochondrial *cox*1 gene showed that *P*. *crassamura* and *P*. *ornamentata* are unique species residing in subclade II of ITS Clade 6 extending the number of described species and designated taxa in this clade and subclade to 31 and 20, respectively [20,21,47]. Inoculation experiments conducted on one-year-old *J*. *phoenicea* and *P*. *lentiscus* seedlings confirmed that both *P*. *crassamura* and *P*. *ornamentata* are pathogenic, supporting their potential involvement in the severe decline that is currently threatening the Mediterranean maquis vegetation in the La Maddalena archipelago.

*Phytophthora crassamura* was previously identified as *P*. *megasperma* [20,21,27,28]. However, the phylogenetic, morphological and physiological comparison between the *P*. *crassamura* isolates and the ex-type culture of *P*. *megasperma* and a couple of isolates with identical sequences clearly support the separation of *P*. *crassamura*. Amongst the isolates of *P*. *crassamura*, there was considerably higher intraspecific variation in the mitochondrial *cox*1 gene than in the nuclear ITS gene sequences. Since the mitochondrial genome evolves more rapidly than genomic DNA, intraspecific variation may be linked to host plant or geographic location [17]. This is consistent with the two lineages of *P*. *crassamura* coming from two different areas with a similar Mediterranean climate but associated with a different range of hosts, including *J*. *phoenicea* and *P*. *lentiscus* in Italy (this study) and *Banksia* sp., *Malus sylvestris* and *Xanthorrhoea platyphylla* in Australia [21]. The differences between the two lineages suggest that *P*. *crassamura* most likely evolved in a different geographic region with either different lineages having been introduced to Australia and Sardinia or the two separate lineages having emerged from similar founder populations as a result of geographic separation in combination with huge differences of potential host plants in the new habitats. A search in the World Phytophthora Collection (WPC) and other previous publications revealed that isolates with sequences identical to *P*. *crassamura* were previously recorded from a wide range of host plants including *Brassica napus* and *Solanum tuberosum* in Australia (WPC P6820 and P7105), *Prunus* sp. in California [20], *Malus sylvestris* in Oregon (WPC P1679), *Pinus eldarica* in Iran [28] and *Prunus persica* in Italy (WPC P7791 and P7792). Almost all findings of *P*. *crassamura* came from ornamental and horticultural environments and appeared to be linked to the trade of plants-for-planting, which is considered as major pathway of *Phytophthora* species [14,48]. Unfortunately, *cox*1 sequences were not available for these isolates to identify to which lineage they belong.

Based on GenBank accession and WPC data, *P*. *ornamentata* has not been isolated elsewhere in the world. Because very little is known about long-term impact of this pathogen on Mediterranean maquis ecosystems, future precautionary measures should be undertaken to prevent and limit its spread. *Phytophthora ornamentata* is homothallic and forms ornamented oogonia, which is a feature of several species from Clades 5 and 7 and two other Clade 6 species, *P*. *gibbosa* and *P*. *missisippiae* [21,47]. Unlike many other taxa from Clade 6 which have abandoned their sexual stage in favor of rapid and abundant asexual multiplication via zoospores in order to compete as saprotrophs and opportunistic pathogens with the multitude of other oomycetes in waterbodies and wet soils [21], *P*. *ornamentata* and *P*. *crassamura* are fully self-fertile and produce oospores with high wall indices (0.63 ± 0.08 and 0.57 ± 0.04, respectively) suggesting that they might have evolved in a dry climate or environment. This survival mechanism has previously been suggested for other *Phytophthora* species that thrive in seasonally dry soils like *P*. *arenaria*, *P*. *elongata*, *P*. *multivora*, *P*. *pachypleura* and *P*. *quercina* [49–51].

The recovery of nine *Phytophthora* species from maquis vegetation and of *P*. *parvispora* from *A*. *unedo* [8] and of *P*. *cinnamomi*, *P*. *cryptogea*, *P*. *gonapodyides*, *P*. *psychrophila* and *P*. *quercina* from *Q*. *ilex* [7,9] constitutes an unusually high diversity of *Phytophthora* species for a small area like the La Maddalena archipelago. This raises questions about the mode of primary introduction and subsequent spread of these pathogens. Movement of living plants by human activities is now generally accepted to be the major pathway of introduction of *Phytophthora* species [48,52,53]. Previous records of some *Phytophthora* species found during this study only from nursery environments in Europe (i.e. *P*. *asparagi* and *P*. *crassamura*) or from distant geographic areas (i.e. *P*. *bilorbang* and *P*. *melonis*), and the widespread planting of exotic plant species such as *Acacia cyanophylla* and *Eucalyptus camaldulensis* for coastal dune protection and restoration over decades suggest infested nursery stock as the primary pathway of *Phytophthora* spp. to the National Park of La Maddalena. The prevalence of dieback symptoms along slopes downhill of roads and trekking paths in all three investigated islands and the fact that decline symptoms were more severe and widespread on the most frequented island Caprera suggest that following their introduction spread of *Phytophthora* spp. across the islands was mainly driven by movement of infested soil attached to tires of cars and bicycles and hiking boots as shown before for ecosystems in Australia [54].

The eradication of *Phytophthora* species once they are established in a new environment is often very difficult, if not impossible, to achieve. However, a number of strategies should be undertaken to mitigate the impact of these pathogens in natural ecosystems. Several studies demonstrated that treatments with fungistatic chemicals such as phosphorous acid (phosphite) provide effective results in controlling *Phytophthora* species in natural ecosystems in Australia [55–57]. Other actions should include the implementation of diagnosis and mapping systems, strict hygiene monitoring activities in highly infested areas, measures to prevent the introduction and spread of *Phytophthora* species including production and distribution of non-infested nursery stock for new plantings, boardwalks in highly infested areas and information and guidance of visitors. Stakeholder engagement, and education and training programs for practitioners should also be given priority. All of these activities together are fundamental for the conservation of biodiversity and social benefits these unique ecosystems provide.

## Supporting Information

S1 Fig The distribution and numbers of isolates of *Phytophthora* species identified during this study from Mediterranean plants and river water. (TIFF).
(TIF)

S2 Fig Mean radial growth rates of *Phytophthora crassamura*, *P*. *megasperma* and *P*. *ornamentata* on Carrot Agar at seven different temperatures. (TIFF).
(TIF)

S1 Table Comparison of variable sites in ITS and *cox*1 gene regions between *Phytophthora megasperma* and *P*. *crassamura*. (DOCX).
(DOCX)
