# Supplementary figures and images for: Diversity of Phytophthora Species from Declining Mediterranean Maquis Vegetation, including Two New Species, Phytophthora crassamura and P. ornamentata sp. nov
Source: PLoS One. 2015 Dec 9;10(12):e0143234. doi: 10.1371/journal.pone.0143234 (PMC4674107; doi:10.1371/journal.pone.0143234)

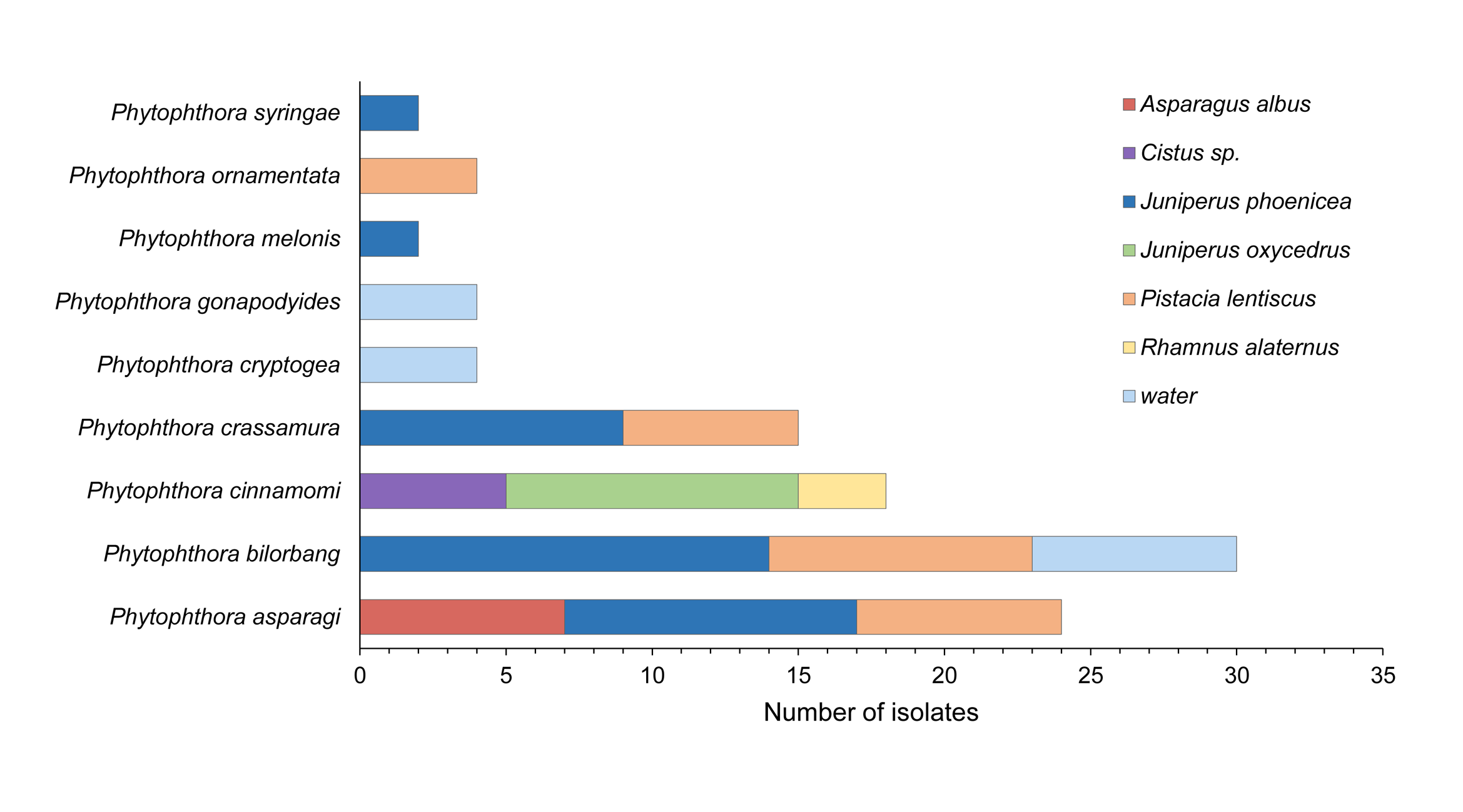

Supplement: S1 Fig — (TIFF). (TIF) [file pone.0143234.s001.tif]

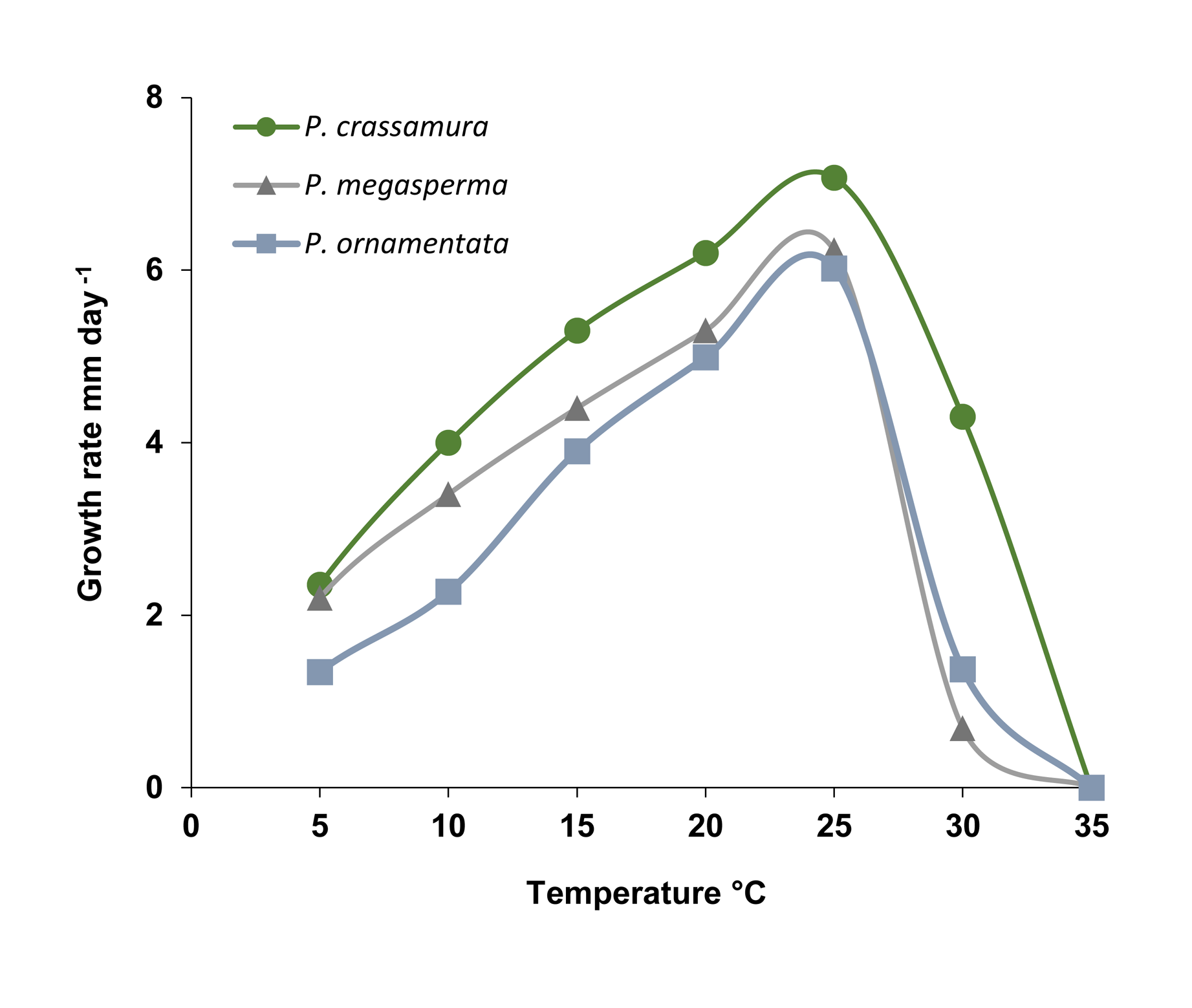

Supplement: S2 Fig — (TIFF). (TIF) [file pone.0143234.s002.tif]
