# Supplementary material for: Diversity of Phytophthora Species from Declining Mediterranean Maquis Vegetation, including Two New Species, Phytophthora crassamura and P. ornamentata sp. nov
Source: PLoS One. 2015 Dec 9;10(12):e0143234. doi: 10.1371/journal.pone.0143234 (PMC4674107; doi:10.1371/journal.pone.0143234)
Supplement: S1 Table — (DOCX). (DOCX) [file pone.0143234.s003.docx]

**S2 Table. Comparison of variable sites in ITS and *cox*1 gene regions between *Phytophthora megasperma* and *P. crassamura*.**

| **Culture no.** | **ITS** | | | | |  | Cox1 | | | | | | | | | | | | | | | | | | | | | | | | | | | | | | | | | | | | | |
| --- | --- | --- | --- | --- | --- | --- | --- | --- | --- | --- | --- | --- | --- | --- | --- | --- | --- | --- | --- | --- | --- | --- | --- | --- | --- | --- | --- | --- | --- | --- | --- | --- | --- | --- | --- | --- | --- | --- | --- | --- | --- | --- | --- | --- |
|  | 102 | 438 | 631 | 694 | 739 |  | 9 | 30 | 57 | 59 | 81 | 120 | 186 | 204 | 264 | 270 | 363 | 384 | 387 | 426 | 438 | 477 | 480 | 507 | 537 | 567 | 570 | 576 | 579 | 588 | 621 | 663 | 681 | 705 | 741 | 771 | 819 | 849 | 963 | 984 | 1002 | 1032 | 1068 | 1095 |
| CBS 402.72 | T | C | G | A | G |  | C | A | C | A | T | C | C | C | C | G | A | A | T | C | C | T | C | A | T | C | T | G | G | A | C | C | A | T | A | T | G | G | T | A | C | C | T | G |
| **PH178** | G | C | G | G | G |  | C | A | C | A | T | C | C | C | A | G | A | A | T | C | C | T | T | A | T | C | T | G | G | A | C | C | A | T | A | T | G | G | T | C | C | C | T | G |
| **PH192** | G | C | G | G | G |  | C | A | C | A | T | C | C | C | A | G | A | A | T | C | C | T | T | A | T | C | T | G | G | A | C | C | A | T | A | T | G | G | T | C | C | C | T | G |
| **CBS 140357** | G | T | A | G | A |  | C | A | T | A | A | C | C | T | A | A | G | T | G | T | T | A | T | T | A | T | A | T | G | C | T | T | C | C | T | T | A | T | A | A | C | T | A | A |
| **PH094** | G | T | A | G | A |  | C | A | T | A | A | C | C | T | A | A | G | T | G | T | T | A | T | T | A | T | A | T | G | C | T | T | C | C | T | T | A | T | A | A | C | T | A | A |
| **PH170** | G | T | A | G | A |  | C | A | T | A | A | C | C | T | A | A | G | T | G | T | T | A | T | T | A | T | A | T | G | C | T | T | C | C | T | T | A | T | A | A | C | T | A | A |
| **PH171** | G | T | A | G | A |  | C | A | T | A | A | C | C | T | A | A | G | T | G | T | T | A | T | T | A | T | A | T | G | C | T | T | C | C | T | T | A | T | A | A | C | T | A | A |
| DDS3432 | G | T | A | G | A |  | C | T | C | A | A | T | C | T | A | A | G | T | G | C | C | C | T | T | A | T | A | T | A | C | T | T | C | C | T | C | A | T | T | A | T | T | A | A |
| VHS17183 | G | T | A | G | A |  | T | T | C | T | A | T | T | T | A | A | G | T | G | C | C | C | T | T | A | T | A | T | A | C | T | T | C | C | T | C | A | T | T | A | T | T | A | A |
| IMI389741 | G | T | A | G | A |  |  |  |  |  |  |  |  |  |  |  |  |  |  |  |  |  |  |  |  |  |  |  |  |  |  |  |  |  |  |  |  |  |  |  |  |  |  |  |
